# Randomised trials comparing different healthcare settings: an exploratory review of the impact of pre-trial preferences on participation, and discussion of other methodological challenges

**DOI:** 10.1186/s12913-016-1823-6

**Published:** 2016-10-19

**Authors:** Mark S. Corbett, Judith Watson, Alison Eastwood

**Affiliations:** 1 Centre for Reviews and Dissemination, University of York, Heslington, York, YO10 5DD UK; 2 York Trials Unit & NIHR Research Design Service Yorkshire & the Humber, University of York, Heslington, York, YO10 5DD UK

**Keywords:** Healthcare settings, Service delivery, Patient preference, Preference trials, Randomised trial

## Abstract

**Background:**

We recently published a systematic review of different healthcare settings (such as outpatient, community or home) for administering intravenous chemotherapy, and concluded that performing conventionally designed randomised trials was difficult. The main problems were achieving adequate trial accrual rates and recruiting a study population which adequately represented the target population of interest. These issues stemmed from the fact that potential participants may have had pre-trial perceptions about the trial settings they may be allocated; such preferences will sometimes be strong enough for patients to decline an invitation to participate in a trial. A patient preference trial design (in which patients can choose, or be randomised to, an intervention) may have obviated these recruitment issues, although none of the trials used such a design.

**Methods:**

In order to gain a better understanding of the broader prevalence and extent of these preference issues (and any other methodological challenges), we undertook an exploratory review of settings trials in any area of healthcare treatment research. We searched The Cochrane Library and Google Scholar and used snowballing methods to identify trials comparing different healthcare settings.

**Results:**

Trial accrual was affected by patient preferences for a setting in 15 of the 16 identified studies; birth setting trials were the most markedly affected, with between 68 % and 85 % of eligible women declining to participate specifically because of preference for a particular healthcare setting. Recruitment into substance abuse and chemotherapy setting studies was also notably affected by preferences. Only four trials used a preference design: the proportion of eligible patients choosing to participate via a preference group ranged from between 33 % and 67 %.

**Conclusions:**

In trials of healthcare settings, accrual may be seriously affected by patient preferences. The use of trial designs which incorporate a preference component should therefore strongly be considered. When designing such trials, investigators should consider settings to be complex interventions, which are likely to have linked components which may be difficult to control for. Careful thought is also needed regarding the choice of comparator settings and the most appropriate outcome measures to be used.

## Background

Although it may seem self-evident that the physical environment of healthcare facilities has the potential to affect health outcomes, only quite recently has there been wide recognition that well-designed physical settings may play such an important role. Research evidence in this area (termed ‘evidence-based design’) has shown that the design of hospital physical environments may influence a range of patient health outcomes; staff outcomes; treatment durations; medication requirements; and may reduce patient, family and staff stress [1].

However, the effect of healthcare settings-the facilities where health interventions are delivered-may often not be evaluated. This may, in part, be due to deficiencies in knowledge and skills about how valid assessments should be performed, and also what should be evaluated [2]. In the UK, the NIHR (National Institute for Health Research) Health Services and Delivery Research (HS&DR) programme funds research to produce evidence on the quality, accessibility and organisation of health services, including evaluations of how the NHS might improve delivery of services [3]. This area of research covers the study of the effect of different healthcare treatment settings.

We (MC and AE) were part of a team which published a HS&DR-funded systematic review which evaluated the clinical- and cost-effectiveness of different healthcare settings for administering intravenous chemotherapy. We studied the effect of home, community, and outpatient settings on a range of outcomes, which were mostly patient-reported outcomes such as quality of life, preference, satisfaction and social functioning. From the trials identified in the systematic review it was apparent that performing randomised trials which compared settings was difficult, particularly in terms of achieving adequate trial accrual rates and recruiting a study population which adequately represented the target population of interest [4].

The inherent nature of settings as interventions means that potential participants may be likely to have pre-trial perceptions (opinions and likely preferences) about the trial settings they may be allocated. For example, some patients may feel anxious about the prospect of receiving treatment in a hospital setting and would rather be treated at home, while others may feel the hospital setting will provide safety and reassurance. These preferences will sometimes be strong enough for eligible patients to decline an invitation to participate in a trial. When performing randomised trials of most kinds of health intervention-though by no means all [5]- this particular type of recruitment problem seems unlikely to result in significant recruitment difficulties. This is because patients typically have little or no experience (real or vicarious) on which to form prior perceptions about at least one of the interventions being evaluated in the trial. It would therefore not be easy for patients to relate the potential benefits and harms of *all* the interventions due to be studied (and presented in a participant information sheet) to themselves as individuals. So, for many types of intervention, the presentation of information to prospective participants which explains the genuine uncertainty about which intervention might be best, should minimise non-participation rates due to preferences. However, the accrual data from the trials included in our chemotherapy setting systematic review suggested that this may not be the case for setting trials. Indeed, it is likely that some patients may decide not to participate *before* reading a participant information sheet.

In our systematic review of chemotherapy settings we concluded that the populations in many of the trials were likely to have been over-represented by hospital-averse (or home-inclined) patients, and under-represented by patients who were keen to receive hospital-based (outpatient) chemotherapy (since the outpatient setting was the only standard of care available to non-participants in nearly all of the trials). These self-selection bias and patient accrual problems appear difficult to overcome by using conventional randomised trial designs. A design which might address such problems is the patient preference trial, of which there are four major types: the Brewin and Bradley design, the comprehensive cohort, the Wennberg design and the Rucker design [5]. The comprehensive cohort design has been used where it is considered that patient preferences may introduce bias if conventional randomisation were to be used [6]. It essentially involves nesting an RCT within a larger observational cohort of patients: ambivalent patients are randomised, and patients with preferences receive their preferred intervention. All (consenting) patients are then followed up. Efficacy estimates would result from the randomised component of the study and any additional influence of motivational factors could be studied by comparing patients randomised to a particular setting with those who *chose* that same setting [7]. In our systematic review, none of the home chemotherapy trials incorporated a preference design.

Conventionally-designed randomised trials investigating the possible effect of a healthcare setting may therefore give rise to small cohorts of participants with results which have limited relevance, or generalisibility, to other populations (i.e. limited external validity), particularly when the combination of pre-trial preferences and subjective patient-reported outcomes arises. Furthermore, as intervention blinding (masking) is not possible in setting trials, patients randomised to their least-preferred option (often the standard care setting) may be more likely to withdraw from the trial, due to the disappointment of not being allocated the newer (or more appealing) setting. This kind of patient reaction to treatment allocation is often termed resentful demoralisation [8]. In light of the findings in our systematic review, and in order to gain a better understanding of the prevalence and extent of these preference and recruitment issues, we undertook an exploratory review of settings trials in any area of healthcare treatment research. While examining these trials we also sought to identify any other setting-related methodological challenges which may be useful to document to help inform the planning and design of future trials. The importance of a consideration of the study designs used in this area of research is particularly relevant, given the call from NHS England’s Chief Executive for changes in service delivery to be tested as rigorously as new treatments [9].

## Methods

We began by searching The Cochrane Library and Google Scholar for relevant studies (or reviews which might include relevant studies). This review was exploratory and search terms were not pre-defined; searching was an evolving, iterative process which utilised search terms such as ‘setting’, ‘home’, ‘community’, ‘home-based’ and ‘inpatient versus outpatient’ (and vice versa). Snowballing methods-such as pursuing references of references and using Google Scholar’s citation search facility-were then used to identify further studies. This has been shown to be a particularly efficient use of search time in reviews of complex evidence [10]. There were no date restrictions.

We included trials where a study objective was to compare the effects of different healthcare settings (i.e. the facilities where health interventions are delivered). For the assessment of the effect of preference on trial recruitment, randomised trials, or studies which consisted of both a randomised cohort and a cohort of patients who chose their treatments, were eligible. The randomisation-only trials had to report the numbers of eligible patients who opted not to be randomised, together with reasons for non-participation. Trials which did not meet these criteria were nevertheless examined for whether any other setting-related challenges with trial conduct were evident. For reasons of practicality, home exercise studies were only considered for cardiac rehabilitation interventions (since a large number of trials with interventions which incorporate home exercise exist). Studies which were stopped early due to recruitment difficulties were eligible.

## Results

### Effect of preferences on accrual and withdrawals for trials not offering a preference option

Table 1 lists the healthcare setting studies identified, with details on how preferences affected patient participation. In addition to intravenous chemotherapy, the clinical areas covered included: opioid dependence, alcohol abuse, cocaine abuse, giving birth, acute pulmonary embolism, deep vein thrombosis, and cardiac rehabilitation.

**Table 1:**
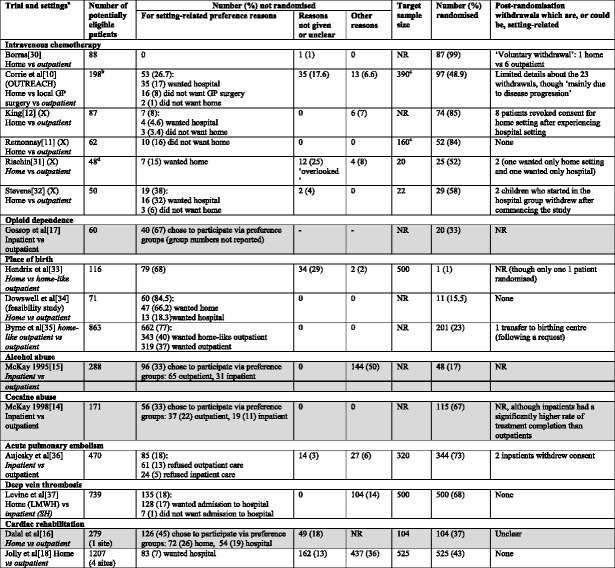
Effect of preferences on accrual and withdrawal in healthcare setting studies reporting reasons for non-participation

Key:

Grey-shaded studies used a patient preference design, all other studies used randomisation only

Numbers in brackets are % of the potentially eligible patients

^a^Italicised settings are those available outside of the trial (where information to assess this is reported)

^b^May be an underestimate of patients actually eligible as ‘clinicians were reluctant to refer patients to the trial’

^c^Trials stopped early

^d^Patients ‘registered in the chemotherapy in the home program’

(X) Cross-over trial

LMWH low-molecular-weight heparin (subcutaneous)

SH standard heparin (intravenous)

Trial recruitment was affected by patient preferences for a setting in 15 of the 16 identified studies. Birth setting trials were the most markedly affected, with between 68 % and 85 % of eligible women declining to participate specifically because of preference for a particular setting. Variation was evident across the intravenous chemotherapy trials with between 0 % and 38 % of eligible patients declining participation due to a setting preference. Recruitment into substance abuse studies was also notably affected by setting preferences with 67 % of opioid abusers, 33 % of alcohol abusers, and 33 % of cocaine abusers opting not to be randomised.

Two trials were stopped early: the OUTREACH trial was stopped due to poor accrual [11] and the Remonnay cross-over trial was stopped because 95 % of participants expressed a preference for home treatment [12]. The latter trial aimed to recruit 160 patients but was stopped when only 52 had been recruited; data from 10 patients who did not participate because they did not want home treatment were seemingly not considered when interpreting the 95 % preference result which triggered the trial to be stopped. It was also unclear how many patients were not invited to participate due to lack of physician consent (which was required as an inclusion criterion) [12]. Clinician views and preferences certainly had some impact on accrual in the OUTREACH trial; the trial authors stated that despite support from clinical colleagues at the trial design stage, in practice clinicians were reluctant to refer patients to the trial, with patient (and staff) safety being a key concern [11].

In contrast to the data on patient accrual into trials, the attrition of patients due to setting preferences did not generally appear to be a problem. Although the reporting of withdrawals was limited in several trials, only one trial reported notable numbers of post-randomisation withdrawals (11 %) for setting reasons [13].

### Effect of preferences on accrual and withdrawals for trials using a preference design

Of the 16 healthcare settings studies identified, only four used a patient preference design in which patients could either opt for randomisation, or for their choice of setting (the shaded studies in Table 1) [14–17]. The proportion of eligible patients choosing to participate via a preference group ranged from between 33 % and 67 %. Some advantages of this study design are illustrated by comparing the two cardiac rehabilitation studies in Table 1: one used conventional randomisation alone [18] and one used a comprehensive cohort design [16]. Both trials were performed in England, recruiting around the same time (between 2002 and 2004 [18], and between 2000 and 2003 [16]). Although both studies *randomised* a similar proportion of eligible patients (around 40 %), the comprehensive cohort study recruited a further 45 % of eligible patients by giving them a choice of setting. The comprehensive cohort trial recruited 82 % of eligible patients compared with 43 % in the trial offering only randomisation. In the latter trial, 28 % of eligible patients ‘did not wish to take part in a research study’. A further advantage of the comprehensive cohort design was the lack of self-selection bias: 7 % of eligible participants in the randomisation-only trial did not participate because they wanted the hospital setting, which was standard care [18]. It is possible that this trial may have had an inflated proportion of patients (at baseline) who preferred the home setting (since participating in the trial was the only way of receiving home treatment). However, in some areas of clinical research even the use of a preference trial may still not prevent the recruitment of a narrower population than desired. This was evidenced by the trial of rehabilitation in male alcoholics: half the eligible patients ‘refused participation in research’ [15].

### Other methodological challenges associated with setting studies

Our exploratory review also found evidence suggesting that the following issues should be considered when planning a setting study.

#### Choice of outcome measures

The choice of outcome assessment measures to be used may warrant additional thought (beyond the considerations needed when evaluating conventional healthcare interventions). Some of the outcome measures available to investigators studying healthcare settings may have only been used previously to evaluate therapeutic interventions, and may therefore not be sensitive enough to detect the benefits associated with a setting. For example, across the home chemotherapy trials, the available quality of life tools tended to focus heavily on physical functioning, rather than on issues such as the time and energy available to patients [4].

Other key outcomes which are often evaluated in setting trials are patient satisfaction and patient preference (i.e. *post*-trial preference). Assessing satisfaction with childbirth settings has been reported as being difficult; satisfaction is determined by a wide variety of factors, so reducing it to a single ordinal outcome may be meaningless [19]. Depending on the study in question, decisions will therefore need to be made on the trade-off between the speed and simplicity of using a single-item measure, and the useful detail provided by more time-consuming multi-item questionnaires [20]. Where patient preference is deemed an important outcome, a study design with a cross-over component should be considered-wherever feasible-since each patient should (theoretically) experience both settings. However, cross-over designs should only really be used for studying patients with relatively stable disease states. Although preferences were studied in many of the home chemotherapy cross-over trials, only one trial investigated *strength* of preference, which proved to be an important assessment: around a third of patients changed their setting preference when they were told their preferred setting was to involve an extra hour of waiting [13]. Results from trials which do not consider strength of preference may therefore have limited use. With these examples in mind, the collection of qualitative patient data should strongly be considered to help evaluate the full range of benefits that different settings may offer. Qualitative data generated from interviews with patients and healthcare professionals before and after a trial can also provide valuable insight regarding barriers to recruitment as well as patients’ healthcare priorities [11].

#### Consideration of settings as complex interventions

Complex interventions are characterised according to several criteria including the number of interacting components, the number and difficulty of behaviours required by those delivering or receiving the intervention, and the degree of intervention flexibility or tailoring permitted [21]. Organisational and care parameters are very likely to form important intervention components when settings are studied. The individual effects of the different, yet interacting components of a setting intervention can be difficult to elucidate. It is therefore likely that most healthcare settings should be considered complex interventions when being evaluated in a trial.

This complexity could make evaluation of any ‘setting effect’ problematic: some investigators may even need to consider whether attempting to study the setting will be viable at all. The following example illustrates how different staff attitudes across settings can have implications for the conduct and results of a trial. An RCT of inpatient versus outpatient opioid detoxification was undertaken because previous trials had methodological limitations-the key one being that different medication regimens had been used in each setting, so the opportunity to study the impact of setting on the likelihood of success had been missed [22]. The newer trial therefore aimed to administer the same medical treatment regimen, for the same period, in an inpatient and an outpatient setting. The same clinical protocol was used for inpatient and outpatient staff, although all staff were given some flexibility in administering the protocol (clinicians could increase the period of full-dose lofexidine by up to 7 days, if clinically indicated). However, at the end of the trial, the outpatient group had received a significantly longer mean medicated period than the inpatient group (17.9 days versus 11.2 days) which was linked to the greater flexibility applied by the outpatient staff. Furthermore, although the protocol required clinicians to terminate the detoxification if a patient tested positive for opioids, cocaine, amphetamine, or unprescribed benzodiazepines, no guidance was provided for cannabis. This led to an unanticipated difference in practice with outpatient nurses routinely ignoring positive cannabis test results, and inpatient staff adopting a strict zero-tolerance approach to all illicit drugs. Other medication differences may have arisen due to the fact that inpatients were supervised in taking all of their medication whereas outpatients were not. Although attempts to control for possible confounders are commendable, this examples suggests this approach should nevertheless be tempered by an acceptance that setting interventions have multiple components which may be inherently linked and may be difficult to control for.

#### Choice of comparator settings

Another issue to consider when designing a setting trial is how ‘standard’ or ‘usual’ the usual care setting is and how likely it is to vary across study sites. New healthcare settings should only be trialled in locations where there appears to be a need. The relevance of this issue was exemplified in a trial of intermediate care clinics for diabetes (ICCD, which are community-based) which were compared with usual GP care (with referral to secondary care as required) [23]. This was a cluster randomised trial (randomising 49 GP practices) performed across three English primary care trusts. The trial had recruitment problems, with GPs not referring enough patients: only 16 % of those eligible were recruited. One of the reasons for this was the variation in the amount of referrals made by practices and professionals. Those making a higher number of referrals tended to view intermediate care clinics as a higher level of care, while those making few referrals were usually from practices with significant diabetes expertise and skills and were therefore less likely to regard intermediate care as offering more than could be offered in-house [24].

## Discussion

The results from our exploratory review suggest that, in trials of healthcare settings, accrual may be seriously affected by patient preferences. The use of trial designs which incorporate a preference component should be more widely adopted when settings are being trialled, since results from conventional RCTs may have very limited applicability to wider patient populations. There may also be important consequences of the small sample sizes which often result from conventional RCTs: trials showing no effect may simply be underpowered to detect effects which might truly exist, or trials with statistically significant results may in fact be reporting chance effects. Investigators planning a trial in this area of research may also need to view the settings as complex interventions which have linked components which may be difficult to control for. Careful consideration may also be needed regarding decisions on which comparator settings and outcome assessment measures might be most appropriate.

The results of a systematic review of preference trials across a broad range of interventions have indicated that although preference groups can sometimes yield different results to randomised groups, self-selected patients do often have similar outcomes to randomised patients [6]. However, those differences in results which were seen in trials in this review were more frequently found to be significant in the smaller studies; this finding is important for our exploratory review since 10 of the 16 studies in Table 1 randomised fewer than 100 patients. Where findings indicate no differences between randomised and preference cohorts, it should also be considered that this may be a reflection of patients choosing a particular treatment for reasons *other* than believing it will be the most effective (in terms of improvements in key trial outcomes). For example, alcohol abusers may prefer inpatient treatment because they want a safe, comfortable place to stay, or they may prefer outpatient treatment as it may not interfere as much with their daily routines [15]. So, effects on patient-perceived quality of life (such as improved relationships, self-awareness and activities of daily living) may be more important to some patients than the effect on the alcohol and drug related outcomes important to the trial investigator [25].

In our exploratory review very limited data were available on *why* patients had preferences which resulted in the offer of participation being declined. One identified study (not tabulated due to the limited detail on reasons for non-participation) did nevertheless highlight that travel issues may adversely affect recruitment. It was an RCT of inpatient versus outpatient chronic pain management; a post-hoc analysis study, which focussed on the effects of patient preference, found that the high rates of refusal to be randomised resulted from the difficulty in traveling from home to hospital. Travel was more demanding for outpatients (in time and costs) than for inpatients. Recruitment was also affected by an unanticipated predominance of patients referred from distant locations; patients living further from the treatment unit were found to be less likely to agree to randomisation [26].

The common theme linking all the methodological issues discussed in our exploratory review is their potential to affect the external validity of trial results. External validity, also sometimes referred to as applicability or generalisability, is the extent to which a result can be reasonably likely to be replicated when applied to a definable group of patients in a particular clinical setting. Lack of external validity is a common criticism by clinicians of RCTs, systematic reviews and guidelines. However, quantification of external validity can be difficult, requiring clinical rather than statistical expertise and a detailed understanding of the particular clinical condition under study and its management in routine clinical practice [27–29]. Assessments of external validity can prove particularly difficult when the information needed is either poorly defined or not reported. The requirement for providing sufficient details on intervention protocols may be especially important as complex interventions may work best if tailored to local circumstances, rather than being completely standardised; clarity in the reporting of how much change or adaptation is permissible is therefore desirable [21]. Both the complexity of the components of setting interventions, and the variability in how patients are recruited (which ultimately causes variability in *who* is recruited) has implications for how practicable it may be for the trialled interventions to be replicated by other organisations.

### Implications for future studies

It appears likely that most of the RCTs identified in our study would have benefitted from using a preference design, although it was unclear why so few of the studies actually gave patients the option of choosing their setting. Perhaps it was due to a lack of knowledge of the existence of such designs, or a fear of straying from the RCT gold standard; the use of less well-known designs may lead to difficulties when acquiring funding, or approvals from ethics or other regulatory committees. Our hope is that in the future, both setting trialists and funders might consider different, arguably more appropriate, methodological approaches than those offered by conventional randomised trial designs. Regardless of the study methods used by investigators, the importance of performing feasibility studies in this area of research cannot be over-stated. Furthermore, any subsequent larger studies should begin with a pilot phase.

In addition to potentially offering improved trial accrual and external validity, patient preference trials may produce more useful estimates of likely rates of uptake of the different settings to help inform future service provision. They may also provide enough data to more clearly identify any setting-related safety issues (which appeared to be one of the key clinician concerns about the implementation of a home or community chemotherapy service [11]). Larger studies might also enable useful assessments to be made of whether setting-related issues which are important to patients vary according to patient characteristics. For example, for patients receiving chemotherapy, waiting times may be more important for patients who are working, whereas transport issues may be more important for elderly patients.

### Limitations

Being exploratory, our review does have limitations. The purpose of the study was to identify challenges and issues which may sometimes be encountered in setting trials in order that they might be minimised in future trials. We did not aim to comprehensively and systematically identify all setting trials, and accept that some relevant studies will not have been identified. Nevertheless, a strength of this study is that we did consider studies from any type of clinical setting in order to try and detect a range of methodological issues. Disappointingly, but perhaps unsurprisingly, our assessment of the impact of preferences on trial recruitment was constrained by the limited reporting of what happened to patients before they were randomised. Many trials did not report adequate details on eligible patients who were not randomised, which limited the number of trials available to us for studying the recruitment outcomes reported in Table 1. Although the CONSORT guidelines (for reporting parallel-group randomised trials) state that the number of patients assessed for eligibility should be reported, it makes little reference of the numbers of eligible patients who were not randomised, and suggests that measures of external validity are arguably less important than the other flow diagram counts [30]. We think that in this area of study the reporting of data to inform external validity is very important. The lack of such data in trial reports may not necessarily be due to limited reporting, but might instead be due to poor trial data acquisition and collation methods.

## Conclusions

In trials of healthcare settings, accrual may be seriously affected by patient preferences. The use of trial designs which incorporate a preference component should therefore strongly be considered. Investigators should consider the implications of the fact that many settings are likely to be complex interventions, which have linked components which may be difficult to control for. When planning setting trials, careful thought is also needed regarding the choice of comparator settings and the most appropriate outcome assessment measures to be used.

## Abbreviations

CONSORT: Consolidated standards of reporting trials
GP: General practitioner
HS&DR: Health services and delivery research
ICCD: Intermediate care clinics for diabetes
NHS: National health service
RCT: Randomized controlled trial
